# Representativeness, diversity, relevance, and participation: a review of the largest Abrasco Congress in history

**DOI:** 10.1590/0102-311XEN014626

**Published:** 2026-04-10

**Authors:** Rômulo Paes-Sousa, Fabiana Damásio

**Affiliations:** 1 Instituto René Rachou, Fundação Oswaldo Cruz, Belo Horizonte, Brasil.; 2 Fiocruz Brasília, Fundação Oswaldo Cruz, Brasília, Brasil.

In 2025, the Brazilian Association of Collective Health (Abrasco, acronym in Portuguese) celebrated its 46th anniversary - nine years older than the Brazilian Unified National Health System (SUS, acronym in Portuguese) itself. From the outset, Abrasco has acted as a catalyst for change, bringing together key actors and ideas essential to achieving a universal, participatory, comprehensive system committed to equity. Its four scientific congresses function as major synthesis moments for the field’s academic and political production.

Collective Health in Brazil comprises a broad educational network: 102 graduate programs distributed across the country. From 2021 to 2024, 5,901 master’s students and 1,851 PhD students completed their program. Additionally, Brazil has 24 undergraduate programs in Collective Health. The 14th Brazilian Congress of Collective Health, held in Brasília from November 28 to December 3, 2025, provided an opportunity to reflect on and celebrate the field’s growth.

The 14th Abrasco Congress established itself as the largest and most diverse in history, reflecting the strength and vitality of Collective Health in Brazil. It included delegations from all Brazilian states: 10,861 registered participants and 7,655 attendees, 72.5% of whom were women. Affirmative action policies supported 1,501 participants. The program featured 148 roundtables, 5 major debates, and 147 pre-congress activities. A total of 4,561 electronic posters were presented, along with 1,698 papers in 189 coordinated oral communication sessions, 869 papers in oral communication sessions, and 30 papers in sessions using alternative formats.

Its theme - *Democracy, Equity, and Climate Justice: Health and Addressing the Challenges of the 21st Century* - expressed a commitment to debating and seeking solutions to three major contemporary issues:

(1) Brazilian institutions have resisted anti-democratic offensives that extend far beyond the failed coup attempt of January 8, 2023. The foundations of democracy are attacked daily. Brazil’s scientific organizations have played an important role in sustaining the country’s resilient, though dysfunctional, democracy.

(2) Collective Health has contributed to advancing knowledge about inequities, both in their most explicit forms and in less visible manifestations. Social public policies shaped by Collective Health researchers, practitioners, and advocates seek to incorporate the fight against inequities into their objectives.

(3) Extreme climate events affecting the country - from the South to the Brazilian Amazon - have caused profound social, economic, and health impacts, especially among the most vulnerable populations. Consequently, health inequities deepen in the most affected areas. These crises include water shortages and wildfires associated with prolonged droughts and criminal activity. They demand flexible, consistent, and effective public policies for disaster prevention and adaptation to global warming - challenges exacerbated by the weakening of environmental legislation in Brazilian National Congress.

The overall tone of the 14th Abrasco Congress presentations acknowledged that the current political moment is promising for Collective Health, despite being marked by significant challenges. Over the nearly three years of the current administration, an imbalance among the government branches has limited the Executive branch’s capacity to act. As a result of coalition presidentialism ^1^, influential forces in Brazilian National Congress - not aligned with the program that prevailed in the 2022 elections - now occupy ministries without relinquishing budgetary capture practices inherited from the previous administration. The magnitude of resources involved in parliamentary amendments and their mandatory execution restrict the Executive’s planning capacity, penalize the health sector, and lead to resource allocation against the SUS principles.

Regardless, the coming years will require a higher standard of federal management for the SUS. With its foundations restored, it is time to address structural deficits, modernize competencies, combat underfunding, and confront regional, cross-cutting, and intersectional inequalities.

For Abrasco’s current board (2024-2027), the 14th Abrasco Congress represented its most significant management commitment. It was a successful political and scientific gathering that addressed the main priorities established in its electoral platform: democracy, equity, sustainability, strengthening the SUS, scientific and technological sovereignty, and socially grounded education.

The 14th Abrasco Congress was an exceptional moment of institutional strengthening for Abrasco, reaffirming its historic values in favor of transformation, independence, transparency, and democracy.

Participants at the Congress held in Brasília sent a clear message: it is time to rethink practices and ways of life - including within academia. Only through social inclusion, mobilization, and unity among democratic forces in Brazil and around the world can we achieve a better future for all.
