# Supplementary material for: Representativeness, diversity, relevance, and participation: a review of the largest Abrasco Congress in history
Source: Cad Saude Publica. 2026 Apr 10;42:e00014626. doi: 10.1590/0102-311XEN014626 (PMC13078782; doi:10.1590/0102-311XEN014626)
Supplement: Translation

# Translation: Representatividade, diversidade, relevância e participação: um balanço do maior Abrascão da história

**DOI:** 10.1590/0102-311XPT014626

**Authors:** Rômulo Paes-Sousa, Fabiana Damásio

**Affiliations:** 1 Instituto René Rachou, Fundação Oswaldo Cruz, Belo Horizonte, Brasil.; 2 Fiocruz Brasília, Fundação Oswaldo Cruz, Brasília, Brasil.

Em 2025, a Associação Brasileira de Saúde Coletiva (Abrasco) completou 46 anos - nove a mais do que o próprio Sistema Único de Saúde (SUS). Desde sempre, a Abrasco foi um catalisador de mudanças, articulando atores e ideias decisivas para tornar viável um sistema universal, participativo, integral e comprometido com a equidade. Seus quatro congressos científicos funcionam como grandes momentos de sínteses da produção acadêmica e política da área.

A área da Saúde Coletiva no Brasil constitui uma rede ampla de formação: são 102 programas de pós-graduação distribuídos pelo país. Entre 2021 e 2024, formamos 5.901 mestres e mestras e 1.851 doutoras e doutores. Há também 24 cursos de graduação em Saúde Coletiva, no Brasil. O 14º Congresso Brasileiro de Saúde Coletiva, que se realizou em Brasília de 28 de novembro a 3 de dezembro de 2025, foi um momento de reflexão e celebração do crescimento da área.

O 14º Abrascão consolidou-se como o maior e mais diverso da história, refletindo a força e a vitalidade da Saúde Coletiva no Brasil. O Congresso contou com delegações de todos os estados do Brasil: 10.861 pessoas inscritas, 7.655 pessoas presentes, sendo 72,5% de mulheres. As ações afirmativas contemplaram 1.501 participantes. Tivemos 148 mesas redondas, 5 grandes debates e 147 atividades no pré-congresso. Foram apresentados: 4.561 pôsteres eletrônicos, 1.698 trabalhos em 189 sessões de comunicação oral coordenada, 869 trabalhos em sessões de comunicação oral e 30 trabalhos em sessões com outras linguagens.

O tema do 14º Abrascão - *Democracia, Equidade e Justiça Climática: A Saúde e o Enfrentamento dos Desafios do Século XXI* - expressou nosso compromisso em debater e buscar soluções para três grandes questões atuais:

(1) As instituições brasileiras têm resistido as investidas antidemocráticas que se estendem muito além da frustrada tentativa de golpe de 8 de janeiro de 2023. Diariamente, o estatuto da democracia é atacado em seus fundamentos. As entidades científicas do Brasil têm cumprido um papel importante na manutenção da resiliente, mas disfuncional democracia brasileira.

(2) A Saúde Coletiva tem contribuído para o conhecimento das iniquidades em suas formas mais explícitas ou nas menos evidentes. As políticas públicas sociais que contam com a contribuição das pessoas da Saúde Coletiva que nelas pesquisam, atuam ou militam buscam inserir em seus objetivos o enfrentamento das iniquidades.

(3) Os eventos climáticos extremos que atingem o país, do Sul à Amazônia, têm causado impactos sociais, econômicos e sanitários profundos, especialmente entre os mais vulnerabilizados. Assim, se aprofundam as iniquidades em saúde nas áreas mais afetadas. Falamos de crises hídricas, incêndios associados a estiagens prolongadas e ações criminosas. Essas situações exigem políticas públicas flexíveis, consistentes e eficientes para prevenção de desastres e adaptação ao aquecimento global - desafios agravados pelo enfraquecimento da legislação ambiental realizado no Congresso Nacional.

A tônica das apresentações no Abrascão reconhece que momento político atual é promissor para a Saúde Coletiva, mas traz desafios importantes. Ao longo desses quase três anos do governo vigente, temos visto um desequilíbrio entre os poderes que tem limitado a atuação do Executivo. Como resultado do presidencialismo de coalizão ^1^, forças influentes no Congresso Nacional, mas que não possuem intimidade com o programa vencedor das eleições de 2022, hoje ocupam ministérios sem renunciar às práticas de sequestro orçamentário herdadas do governo anterior. A dimensão dos recursos envolvidos nas emendas parlamentares e respectiva obrigatoriedade de implementação restringem a capacidade de planejar do Executivo, penaliza a saúde e gera uma alocação de recursos distante dos princípios do SUS.

Apesar disso, os próximos anos colocam diante de nós um patamar mais elevado para a gestão federal do SUS. Com suas bases restabelecidas, é hora de enfrentar déficits estruturais, modernizar competências, combater o desfinanciamento e enfrentar desigualdades regionais, transversais e interseccionais.

O 14º Abrascão foi para a atual diretoria da Abrasco o seu mais importante compromisso de gestão (2024-2027). Foi um bem-sucedido encontro político e científico que cobriu os principais temas firmados em seu compromisso eleitoral, quais sejam: democracia, equidade, sustentabilidade, fortalecimento do SUS, soberania científica e tecnológica, e educação socialmente referenciada.

O 14º Abrascão foi um excepcional momento de fortalecimento institucional da Abrasco, onde reafirmamos os nossos valores históricos em favor da transformação, independência, transparência e democracia.

Os participantes do Congresso realizado em Brasília nos enviaram uma clara mensagem: é tempo de repensar práticas e modos de vida - inclusive dentro da academia. Somente com inclusão social, mobilização e união das forças democráticas do Brasil e de todo o mundo, poderemos ter um futuro melhor para todas e todos.

# Translation: Representatividad, diversidad, relevancia y participación: un balance del mayor Abrascão de la historia

**DOI:** 10.1590/0102-311XES014626

**Authors:** Rômulo Paes-Sousa, Fabiana Damásio

**Affiliations:** 1 Instituto René Rachou, Fundação Oswaldo Cruz, Belo Horizonte, Brasil.; 2 Fiocruz Brasília, Fundação Oswaldo Cruz, Brasília, Brasil.

En el 2025, la Asociación Brasileña de Salud Colectiva (Abrasco, por su sigla en Portugués) cumplió 46 años, nueve años más que el propio Sistema Único de Salud de Brasil (SUS). Desde sus inicios, Abrasco ha sido un catalizador de cambios, articulando a actores e ideas decisivas para hacer viable un sistema universal, participativo, integral y comprometido con la equidad. Sus cuatro congresos científicos funcionan como importantes momentos de síntesis de la producción académica y política del ámbito.

El ámbito de la Salud Colectiva en Brasil constituye una amplia red de formación: existen 102 programas de posgrado distribuidos por todo el país. Entre el 2021 y el 2024, capacitamos a 5.901 docentes y a 1.851 doctoras y doctores. También hay 24 programas de grado en Salud Colectiva, en Brasil. El 14º Congreso Brasileño de Salud Colectiva, que se celebró en Brasilia del 28 de noviembre al 3 de diciembre del 2025, fue un momento de reflexión y celebración del crecimiento de este ámbito.

El 14º Abrascão se consolida como el más grande y diverso de la historia, reflejando la fuerza y vitalidad de la Salud Colectiva en Brasil. El Congreso incluyó delegaciones de todos los estados brasileños: se inscribieron 10.861 personas, 7.655 personas presentes, de las cuales el 72,5% eran mujeres. Los programas de acción afirmativa incluyeron 1.501 participantes. Tuvimos 148 mesas redondas, 5 grandes debates y 147 actividades precongreso. Se presentaron los siguientes trabajos: 4.561 pósteres electrónicos, 1.698 trabajos en 189 sesiones de comunicación oral coordinada, 869 trabajos en sesiones de comunicación oral y 30 trabajos en sesiones utilizando otros lenguajes.

El tema del 14º Abrascão -*Democracia, Equidad y Justicia Climática: Salud y Cómo Afrontar los Desafíos del Siglo XXI-* expresó nuestro compromiso de debatir y buscar soluciones para tres importantes temas actuales:

(1) Las instituciones brasileñas han resistido a ataques antidemocráticos que se extienden mucho más allá del fallido intento de golpe de Estado del 8 de enero del 2023. A diario se atacan los cimientos mismos del estatuto de la democracia. Las instituciones científicas de Brasil han desempeñado un papel importante en el mantenimiento de la resiliente, pero disfuncional democracia del país.

(2) La Salud Colectiva ha contribuido a comprender las inequidades en sus formas más explícitas o en las menos evidentes. Las políticas públicas sociales que se benefician de las contribuciones de las personas de la Salud Colectiva que en ellas investigan, actúan o militan buscan incluir en sus objetivos el afrontamiento de las iniquidades.

(3) Los fenómenos climáticos extremos que afectan al país, desde el sur hasta la Amazonia, han causado profundos impactos sociales, económicos y sanitarios, especialmente entre los más vulnerabilizados. De esta forma, se profundizan las inequidades en salud en las zonas más afectadas. Hablamos de crisis hídricas, de incendios asociados a sequías prolongadas y de actos delictivos. Estas situaciones requieren políticas públicas flexibles, consistentes y eficientes para la prevención de desastres y la adaptación al calentamiento global, desafíos que se ven agravados por el debilitamiento de la legislación ambiental en el Congreso Nacional.

El tema principal de las presentaciones en Abrascão reconoce que el momento político actual es prometedor para la salud colectiva, pero trae desafíos importantes. A lo largo de estos casi tres años del actual gobierno, hemos visto un desequilibrio entre los poderes que ha limitado la actuación del Poder Ejecutivo. Como resultado del presidencialismo de coalición ^1^, fuerzas influyentes en el Congreso Nacional, pero que no tienen proximidad con el programa ganador de las elecciones del 2022, ahora ocupan ministerios sin renunciar a las prácticas de secuestro presupuestario heredadas del gobierno anterior. La magnitud de los recursos involucrados en las enmiendas parlamentarias y su obligatoriedad de implementación restringen la capacidad de planificación del Poder Ejecutivo, penalizan la salud y conducen a una asignación de recursos muy alejada de los principios del SUS.

Pese a ello, los próximos años nos presentan un estándar más elevado para la gestión federal del SUS. Una vez restablecidas sus bases, es hora de afrontar los déficits estructurales, modernizar las competencias, combatir la desfinanciación y afrontar las desigualdades regionales, transversales e interseccionales.

El 14º Abrascão fue para la actual dirección de Abrasco su compromiso de gestión más importante (2024-2027). Fue un encuentro político y científico exitoso que cubrió los principales temas firmados en su compromiso electoral, a saber: democracia, equidad, sostenibilidad, fortalecimiento del SUS, soberanía científica y tecnológica y educación socialmente referenciada.

El 14º Abrascão fue un momento excepcional de fortalecimiento institucional de la Abrasco, en el que reafirmamos nuestros valores históricos a favor de la transformación, la independencia, la transparencia y la democracia.

Los participantes del Congreso celebrado en Brasilia nos enviaron un mensaje claro: es hora de repensar las prácticas y los modos de vida, incluso en la academia. Solo mediante la inclusión social, la movilización y la unidad de las fuerzas democráticas en Brasil y en el mundo podremos tener un futuro mejor para todas y todos.
